# 

**Published:** 1842-04-16

**Authors:** 


☞The EXAMINER makes its appearance to-day, and will be henceforward published, enclosed in a cover. The editors are unwilling to disfigure the body of the work with advertisements, and as very many of their subscribers have expressed a preference for the old dress, they have determined to restore it. The circulation of the journal has greatly increased since the commencement of the new series: It is now sent into twenty-five States, and the three Territories of the Union, Canada, the West Indies, Mexico, and South America, and offers a very desirable medium to the medical schools, booksellers, and others who wish to receive the attention of the profession.

---

☞Owing to the unexpected occurrence of circumstances of a private nature, Dr. Reynell Coates retires from the editorship of the Examiner. He will continue to be a principal writer for the journal, and will still edit the surgical selections.

Published EVERY SATURDAY by J. C. TURNPENNY, N. E. corner of Spruce and Tenth streets.

*Price of Subscription*—Three Dollars a year, or two copies for Five Dollars to the same post office, always in advance. Subscriptions must commence with the beginning of the annual volume. Letters to be addressed, post free, to the Editors of the Medical Examiner.

*Rates of Advertising in the Medical Examiner*—For one insertion of a square of twelve lines, one dollar, and for each additional insertion, half a dollar.

